# Myriocin enhances the clearance of *M. tuberculosis* by macrophages through the activation of PLIN2

**DOI:** 10.1128/msphere.00257-24

**Published:** 2024-06-26

**Authors:** Ximeng Zhang, Guanggui Ding, Xirui Yang, Hailin Lu, Yuzhong Xu, Yunlong Hu, Song Liu, Huihua Zhang, Kaisong Huang, Guofang Deng, Taosheng Ye, Qing Yu, Yi Cai, Shuixiang Xie, Wenfei Wang, Xinchun Chen

**Affiliations:** 1 Guangdong Provincial Key Laboratory of Regional Immunity and Diseases, Department of Pathogen Biology, Shenzhen University Medical School, Shenzhen, China; 2 Shenzhen People’s Hospital, The Second Clinical Medical College, Jinan University, The First Affiliated Hospital, Southern University of Science and Technology, Shenzhen, Guangdong, China; 3 Dornsife College of Letters, Arts and Sciences, University of Southern California, Los Angeles, California, USA; 4 Key Laboratory of Prevention and Treatment of Cardiovascular and Cerebrovascular Diseases, Ministry of Education, Gannan Medical University, Ganzhou, China; 5 Department of Clinical Laboratory, Shenzhen Baoan Hospital, Shenzhen, China; 6 Guangdong Provincial Key Laboratory of Medical Molecular Diagnostics, The First Dongguan Affiliated Hospital, Guangdong Medical University, Dongguan, China; 7 National Clinical Research Center for Infectious Disease, Shenzhen Third People’s Hospital, Southern University of Science and Technology, Shenzhen, China; Washington University in St. Louis School of Medicine, St. Louis, Missouri, USA

**Keywords:** tuberculosis, lipid droplets metabolism, myriocin, PLIN2, host-directed therapy

## Abstract

Myriocin is an inhibitor of *de novo* synthesis of sphingolipids and ceramides. In this research, we showed myriocin could significantly reduce Mtb burden and histopathological inflammation in mice. However, the underlying mechanism remains unclear. RNA-seq analysis revealed a significant increase in gene expression of PLIN2/CD36/CERT1 after myriocin treatment. The reduced bactericidal burden was only reversed after silencing the lipid droplets (LDs) surface protein PLIN2. This suggests that myriocin enhances the ability of macrophages to clear Mtb depending on the PLIN2 gene, which is part of the PPARγ pathway. Indeed, we observed a significant increase in the number of LDs following myriocin treatment.

**IMPORTANCE:**

*Mycobacterium tuberculosis* has the ability to reprogram host cell lipid metabolism and alter the antimicrobial functions of infected macrophages. The sphingolipids, such as ceramides, are the primary host lipids utilized by the bacteria, making the sphingomyelinase/ceramide system critical in Mtb infections. Surprisingly, the antimicrobial effect of myriocin was found to be independent of its role in reducing ceramides, but instead, it depends on the lipid droplets surface protein PLIN2. Our findings provide a novel mechanism for how myriocin enhances Mtb clearance in macrophages.

## INTRODUCTION

Tuberculosis (TB) is a communicable disease caused by the bacterium *Mycobacterium tuberculosis* (Mtb), resulting in 10 million infections and 1.4 million deaths annually (1). Drug resistance, long treatment duration, and medication side effects are the main limitations of traditional tuberculosis drug treatment methods. However, most individuals can suppress Mtb infection and prevent the development of active TB pathology through a balanced metabolic immune response (2–4). The lipid composition performs important functions in interaction between macrophages and Mtb which has evolved multiple strategies to survive intracellularly in macrophages and extracellularly within lipid-rich granulomas (5, 6).

Mtb has the ability to reprogram host cell lipid metabolism to acquire essential nutrients and develop its unique lipid-rich cell wall, which facilitates its prolonged persistence *in vivo* (7). Consequently, Mtb infection disrupts host lipid metabolism and alters the antimicrobial functions of infected macrophages. The field of immunometabolism has emerged to shed light on the connection between macrophage metabolic states and their immunological responses to Mtb infection (8). Recent studies have revealed various roles of the sphingomyelinase/ceramide system (SCS) in manipulating Mtb infections. Utermöhlen et al. demonstrated that SCS is necessary for phagolysosomal fusion in macrophages, an essential aspect of the innate immune response for eliminating intracellular pathogens *M. avium*. Mice deficient in SCS showed increased survival after infection without exhibiting clinical signs of disease (9). Subsequently, a zebrafish model infected with *M. marinum* also exhibited a survival strategy used by mycobacteria that involves SCS. Tumor necrosis factor (TNF) induces the production of reactive oxygen species (ROS), which can rapidly lead to necroptosis through SCS-mediated ceramide production. Additionally, necroptosis results in the release of mycobacteria into the growth-permissive extracellular environment (10). Despite the recognized association between Mtb and host lipids, the underlying mechanism remains elusive (11). Drugs targeted on SCS might reverse the susceptibility to Mtb.

In our study, we investigated the effect of myriocin, an inhibitor of SCS, on Mtb infection. In line with what we expected, myriocin significantly reduced Mtb burden and histopathological inflammation in mice. RNA-seq was established to identify genes that are involved in myriocin-treated macrophages upon Mtb infection. The result showed that LDs surface protein PLIN2 and ceramide transporter protein CERT1 gene expression increased significantly after myriocin treatment. Surprisingly, the antimicrobial effect of myriocin was independent of its role in reducing ceramides. The reduced bactericidal burden was only reversed after silencing the lipid droplets (LDs) surface protein PLIN2 but not CERT1.

LDs are major organelles involved in lipid storage, consisting of a central core of neutral lipids surrounded by a phospholipid monolayer (12). Recent studies have highlighted the essential role of LDs in cellular stress response (13). LDs can trigger the fusion of phagosomes with lysosomes, leading to the release of antimicrobial factors that kill Mtb (14). Based on this, it differs from the traditional understanding that LDs provide nutrients to Mtb and promote its intracellular survival in the host. This suggests an alternative hypothesis that LDs have protein-mediated antimicrobial capabilities and myriocin regulates immune metabolism through the lipid droplet surface protein PLIN2. Indeed, we found a significant increase in the number of LDs following myriocin treatment.

In conclusion, our findings demonstrate that myriocin enhances Mtb clearance in macrophages through the involvement of LDs and the activation of PLIN2. This novel mechanism may serve as a potential target for host-directed therapy (HDT) against tuberculosis.

## MATERIALS AND METHODS

### Mtb culture and infection

Mtb strains (H37Ra, GFP-H37Ra, and H37Rv) were cultured in 7H9 broth (BD Biosciences, San Jose, CA, USA) containing 0.2% glycerol (Sigma-Aldrich, Merck, Darmstadt, Germany), 10% Middlebrook oleic acid-albumin-dextrose-catalase (OADC) enrichment (BD), and 0.05% Tween 80 (Sigma-Aldrich). Cultures were incubated at 37°C with shaking until they reached the mid-logarithmic phase (optical density at 600 nm [OD_600_] of 0.3–0.8) before use in experiments. For all experiments involving mycobacterial infection, bacteria cultured in 7H9 medium were washed once with phosphate-buffered saline (PBS), resuspended in serum-free RPMI 1640 medium, and sonicated for 5 min to obtain a single-cell suspension.

### Cell culture

The human monocytic cell line THP-1 was obtained from the Cell Bank of the Chinese Academy of Sciences (Shanghai, China) and cultured in RPMI 1640 medium (Corning, USA) supplemented with l-glutamine (2 mM) and 10% fetal bovine serum (Gibco, Life Technologies). To differentiate THP-1 cells into macrophages, they were seeded in 12-well plates at a density of 3 × 10^5^ cells/mL and treated with 40 ng/mL phorbol 12-myristate 13-acetate (PMA; Sigma-Aldrich, Merck, USA) for 24 h at 37°C in a humidified atmosphere containing 5% CO2. Differentiated THP-1 cells were maintained in fresh complete RPMI 1640 medium under the same conditions for subsequent experiments.

### Cell viability assay

Cell viability of THP-1 cells treated with various chemicals was assessed using the CCK-8 Cell Proliferation and Cytotoxicity Assay Kit (MCE, Shanghai, China). Differentiated THP-1 cells were seeded in 96-well plates at a density of 5 × 10^4^ cells/well and treated with myriocin, FB1, GW9662, or rosiglitazone at appropriate concentrations for 24 and 72 hours. Cells treated with DMSO served as a negative control. After treatment, CCK-8 reagent (Beyotime, Shanghai, China) was added (10 µL/well), and the cells were incubated for 2 h at 37°C. The formazan dye produced by viable cells was quantified by measuring the absorbance at 450 nm using the EpochTM 2 microplate spectrophotometer. The absorbance at 650 nm was measured as the reference wavelength. Cell viability was calculated according to the manufacturer’s instructions.

### Cell isolation and polarization of human MDM

Leukocyte concentrates from freshly withdrawn peripheral blood of male and female healthy adult volunteers were provided by Shenzhen Third People’s Hospital. Peripheral blood monocytic cells (PBMCs) were separated using dextran sedimentation, followed by centrifugation on lymphocyte separation medium (Histopaque-1077). For isolation of monocytes, PBMC were resuspended in RPMI 1640 containing 10% (vol/vol) heat-inactivated fetal calf serum (FCS), 100 U/mL penicillin, and 100 mg/mL streptomycin in cell culture flasks for 1.5 h at 37°C and 5% CO2 for adherence of monocytes. For differentiation of the monocytes to MDM which were generated by incubating monocytes with 20 ng/mL GM-CSF for 6 days in RPMI 1640 supplemented with 10% FCS, 2 mmol/L glutamine (Biochrom/Merck, Berlin, Germany), and penicillin–streptomycin (Biochrom/Merck) to obtain M0_GM-CSF_MDM (briefly “MDM”).

### CFU analysis of infected cells

Differentiated THP-1 cells (3 × 10^5^ cells/mL) were infected with H37Ra/H37Rv (MOI = 10) for 6 h, washed three times with 1× PBS to remove extracellular bacteria, and then incubated in fresh RPMI 1640 medium with myriocin or FB1 for an additional 72 h at 37°C under 5% CO_2_. For colony-forming unit (CFU) counting, cells were washed three times with 1× PBS, lysed in 1× PBS containing 0.1% SDS for 10 min at room temperature, and serially diluted with 1× PBS. About 50  µL of each diluted sample was plated on 7H10 agar plates and incubated at 37°C for 15–20 days.

### High throughput targeted quantification for metabolites

The sample extracts were analyzed using Waters UPLC I-Class Plus (Waters, USA) equipped with QTRAP 6500 Plus (SCIEX, USA) at the Department of Mass Spectrometry, BGI-Shenzhen. Following the protocol provided by the manufacturer. The MRM (multiple reaction monitoring) method was set under MRM mode, and it includes MRM parent-daughter transition information of target metabolites, collision energy (CE), declustering potential (DP), and retention time. Using skyline a powerful to perform metabolite identification and quantification.

### Confocal microscopy

Differentiated THP-1 macrophages were transfected with PLIN2 or control siRNA for 48 h before infection with GFP-H37Ra (MOI = 10) for an additional 6 h. The cells were then treated with myriocin (5 µM) and incubated for another 24 h. After collection, macrophages were washed two times with PBS, fixed with 4% paraformaldehyde for 15 min, and permeabilized with 0.3% Triton X-100 for 10 min. Lipid droplets were stained using HCS LipidTOX Red Neutral Lipid Stain (Cat #H34476, Invitrogen, USA) at 37°C for 30 min in the dark. Nuclei were stained with DAPI (Leagene, China) for 5 min. Images were acquired using an Olympus FV1000 confocal microscope (NIKON A1R).

### Immunohistochemistry

Lung tissue segments from mice were fixed in 10% buffered formalin (Sigma-Aldrich) and embedded in paraffin. Histologic sections were stained with hematoxylin and eosin (H&E) for evaluation of pathology. Whole microscope slide images were digitally photographed using the NanoZoomer Digital Pathology System (Hamamatsu Photonics). For immunohistochemistry, 3-µm-thick paraffin sections were collected on charged slides, deparaffinized, rehydrated in water, and subjected to antigen retrieval in pH 6.0 citrate buffer for 5 min at 125°C in a pressure cooker. Sections were then incubated with a primary antibody against PLIN2 for 2 h at room temperature. Immunoreactions were visualized using a biotin-free dextran-chain detection system (Envision, DakoCytomation, Glostrup, Denmark) and developed using 3′,3′-diaminobenzidine as the chromogen.

### RNA-Seq and real-time quantitative PCR

Differentiated THP-1 macrophages (5 × 10^5^ cells/well) were cultured in 12-well plates, infected with H37Ra for 6 h, and treated with or without myriocin (5 µM) for 24 h. Total RNA extraction was performed using the RNAeasy Minikit (Qiagen), and sequencing was conducted as previously described (15) With a negative binomial distribution-based model adopted by edgeR, the gene expression levels were normalized among replicates and between groups, and logarithmic transformation of normalized count per million (logCPM) for each gene was used for further comparative analysis. Gene expression was compared between groups using edgeR with quasi-likelihood *F*-tests. The significance level was preset as a multi-testing corrected false discovery rate of 0.05. Gene enrichment analysis shows CD36 and PLIN2 in the PPARγ signaling pathway, potentially associated with sphingolipid metabolism; hence, we chose them along with the CERT1 gene. cDNA synthesis was carried out using an oligo-dT primer and SuperScript II reverse transcriptase (Invitrogen, Carlsbad, CA). Gene expression was measured using SYBR Green-based real-time quantitative PCR. Data were analyzed using the 2^−ΔΔCT^ method with β-actin as the housekeeping gene.

### RNA interference

Differentiated THP-1 macrophages (5 × 10^5^ cells/mL) were transfected with PLIN2, CD36, and CERT1 siRNA (RiboBio; China) using liposomal RNAi MAX (ThermoFisher Scientific, USA). siRNA and liposome complexes were prepared in Opti-MEM (Gibco). After 6 h of transfection, the medium was replaced, and the cells were further cultured for 48 h to allow gene silencing. The knockdown efficiency was assessed by qPCR.

### Western blotting

Differentiated THP-1 macrophages (5 × 10^5^ cells/mL) were cultured in 12-well plates and infected with H37Ra with or without the addition of myriocin (5 µM) for 24 h. Cells were washed with PBS and lysed in radioimmunoprecipitation lysis buffer (Beyotime) supplemented with protease inhibitor cocktail (Roche). Protein concentrations were determined using the BCA protein kit (Beyotime). About 10 µg of cell lysates was loaded, separated by SDS-PAGE, transferred to PVDF membranes (Millipore), and blocked with 5% nonfat milk in PBST containing 0.1% Tween-20 for 1 h at room temperature. Membranes were then incubated overnight at 4°C with primary antibodies against PPARγ (Proteintech; 16643-1-AP, USA), PLIN2 (Abcam; ab108323, USA), and β-actin (Abcam; ab179467, USA). After incubation with HRP-conjugated secondary antibody (Abcam, USA) for 2 h at room temperature, protein bands were visualized using an ECL detection solution (ThermoFisher Scientific, USA). Digital images were acquired using a chemiluminescence imaging system (China Micro).

### Murine TB model

C57BL/6 mice (6–8 weeks old) were infected with H37Rv using a glass cavity inhalation system. On day 14 post-infection, mice received an intraperitoneal injection of myriocin (5 mg/kg/2 days). The control group received only 0.1% DMSO. On day 28, mice were sacrificed, and lung tissue homogenization was performed, followed by 10-fold serial dilutions. The diluted samples were plated on 7H10 agar plates to determine the bacterial counts in the lungs. Hematoxylin and eosin staining was used for histopathological analysis of the lung tissue. Lung lobes were digitally imaged using a nano-zoomer digital pathology system (Hamamatsu Optoelectronics).

### Statistical analysis

Data are presented as mean ± standard deviation (SD) of *n* observations, where *n* represents the number of experiments conducted with separate donors on different days, as indicated. GraphPad Prism 9 software (San Diego, CA) was used for data analysis. Paired *t* test was used for comparing two groups, while one-way analysis of variance (ANOVA) with Bonferroni or Dunnett’s post hoc tests was applied for multiple comparisons, as indicated. The criterion for statistical significance was set at *P* < 0.05.

## RESULTS

### Myriocin enhances Mtb clearance in macrophages involved in lipid metabolism

**Fig 1:**
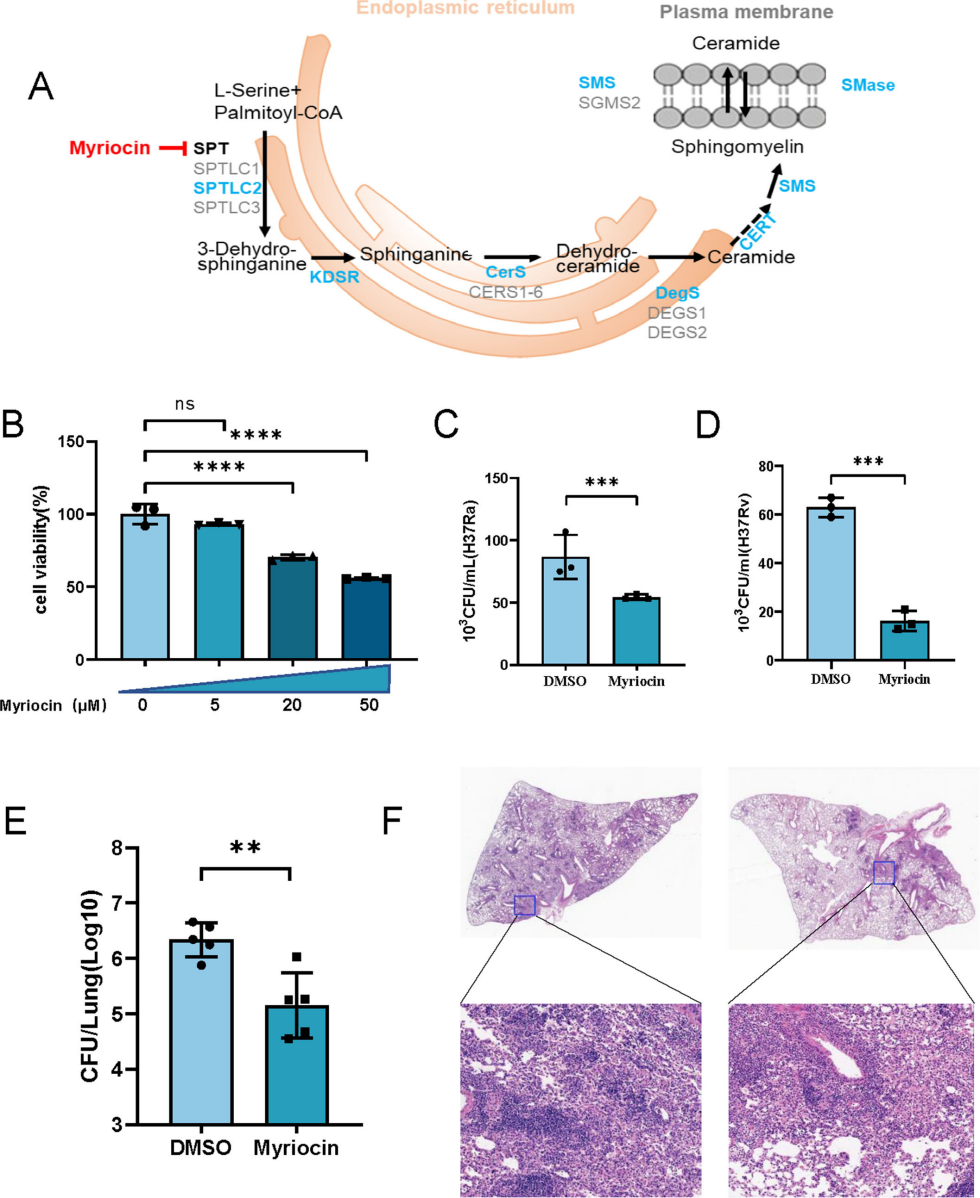
Myriocin reduces bacterial load and pathology *in vitro* and *in vivo*. (**A**) Schematic diagram for *de novo* synthesis of sphingolipids and ceramides. (**B**) Effect of myriocin on cell viability. PMA-differentiated THP-1 macrophages were treated with indicated concentration of myriocin. After 24 h incubation at 37°C, cell viability was assessed by CCK-8 assay. (**C and D**) PMA-differentiated THP-1 macrophages were infected with H37Ra (C) and H37RV (D) (MOI = 10:1) for 6 h. Then extracellular bacteria were removed, after 72 h incubation with myriocin, intracellular CFU was determined. (**E**) C57BL/6 mice (6–8 weeks old) were infected with H37Rv for 14 days. Then the mice were intraperitoneally injected with myriocin (5 mg/kg/2 days) or 0.1% DMSO, respectively. Mice were sacrificed on day 28, tissue homogenization was 10-fold serial dilutions and plated on 7H10 agar plates to determine lung bacterial counts. (**F**) Histopathology of lungs infected with Mtb, stained with hematoxylin and eosin. Outlined areas in main images are enlarged in insets. Data are means ± S.E.M., *n* = 3. Unpaired two-tailed Student’s *t* test was used to analyze the difference between the two groups. The differences among groups were compared using one-way ANOVA followed by Tukey’s multiple comparison test. *P < 0.05, **P < 0.01, ***P ≤ 0.001.

Myriocin is an effective inhibitor of serine-palmitoyl transferase (SPT), which is crucial for *de novo* synthesis of sphingolipids and ceramides (Fig. 1A). To investigate the effect of myriocin on the immune response against Mtb infection, Thp-1 cells were pretreated with or without 5 µM myriocin, which did not affect cell viability both in 24 and 72 h (Fig. 1B; Fig. S1A). The cells were then infected *in vitro* with both the virulent strain H37Rv and the avirulent counterpart H37Ra. The CFU assay revealed a significant reduction in bacterial load after myriocin treatment for both H37Rv and H37Ra (Fig. 1C and D). To validate the role of myriocin in bacterial control *in vivo*, mice infected with H37Rv were treated with myriocin or DMSO. Myriocin treatment significantly reduced bacterial load and ameliorated immunopathology (Fig. 1E and F).

FB1 is also an inhibitor of ceramide synthase, we also found that 5 µM FB1 pre-treatment did not affect cell viability both in 24 and 72 h which can also enhance macrophage clearance ability against Mtb (Fig. S1A and B). To validate the inhibitory effect of myriocin on the sphingolipid pathway. We detected lipid metabolites in the supernatant of cell cultures (Fig. S2A). Both sphingomyelin (SM) and ceramides (Cre) were downregulated after myriocin treatment during Mtb infection (Fig. S2I and J). All these data indicated that sphingolipids and ceramides metabolism are involved in Mtb clearance in macrophages.

### Myriocin induces the clearance of Mtb by macrophage depends on PLIN2 expression

**Fig 2:**
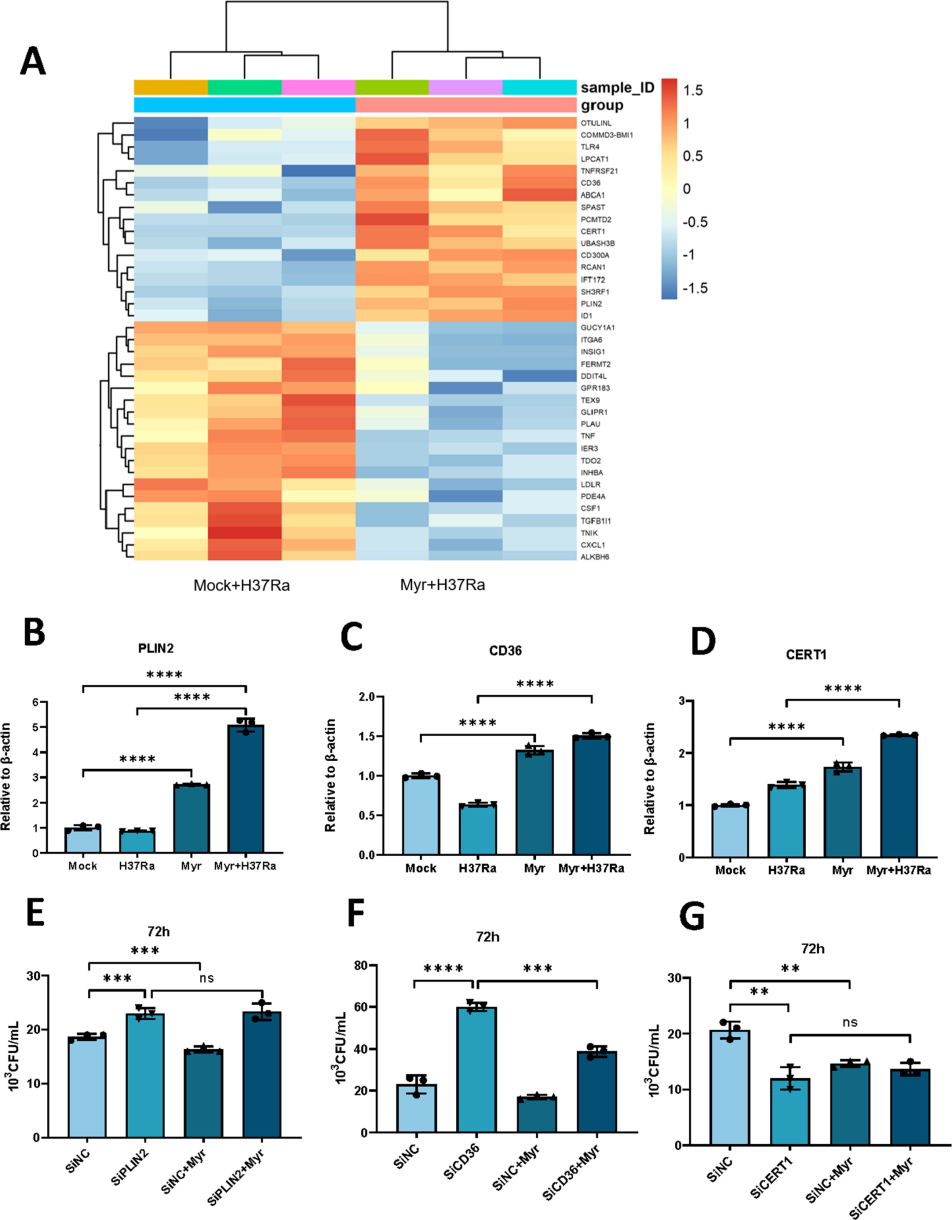
Myriocin induced the clearance of Mtb by macrophage depends on PLIN2 expression. (**A**) Total RNA was extracted and RNAseq analysis was performed. Shown are genes that displayed a significant mean fold change treated or not with myriocin during 24 h infection of H37Ra, *n* = 3. (**B and C**) Analysis of mRNA expression levels of PLIN2 (**B**), CD36 (**C**) with or without myriocin treatment during H37Ra infection or not in PMA-differentiated THP-1 macrophages. Relative mRNA levels are normalized to β-actin, *n* = 3. (**D and E**) PMA-differentiated THP-1 macrophages were transfected with siPLIN2 (**D**), siCD36 (**E**), or control siRNA for 48 h before infection with H37Ra (MOI  =  10:1) for another 72 h and CFU assay was performed, *n* = 3. Data are means ± S.E.M., *n* = 3. The differences among groups were compared using one-way ANOVA followed by Tukey’s multiple comparison test. *P < 0.05, **P < 0.01, ***P ≤ 0.001.

We further investigated the genes involved in lipid storage that may be regulated by myriocin during Mtb infection. RNA-sequencing analysis revealed several upregulated genes associated with lipid metabolism, including PLIN2, CD36, and CERT1, which play a crucial role in ceramide transport (Fig. 2A). We confirmed that myriocin treatment resulted in higher expression levels of these three genes, regardless of Mtb infection (Fig. 2B through D). To confirm the veracity of the RNA sequencing data, we enhanced the research by conducting cell experiments where THP-1 cells were stimulated with H37Rv. Subsequently, we validated the chosen genes from the RNA-Seq data through q-PCR analysis. The outcomes were in alignment with those obtained from H37Ra (Fig. S3A through E). To assess the role of these genes in myriocin-induced Mtb clearance, we conducted a CFU assay after silencing gene expression using siRNA which exhibits excellent knockdown efficiency (Fig. S4A through D). The phagocytic efficiency of the cells remains unchanged after Mtb infection for 6 h (Fig. S4E through G) and the results revealed an elevated bactericidal burden following the silencing of PLIN2 and CD36 in 72 h. However, silencing CERT1 decreases bactericidal burden. Interestingly, only silencing PLIN2 did not lead to a reduction in the bactericidal burden after myriocin treatment, suggesting that myriocin-induced Mtb clearance is dependent on the expression of the PLIN2 gene (Fig. 2D). Conversely, silencing CD36 and CERT1 also reduced the bactericidal burden after myriocin treatment, suggesting that myriocin-induced clearance of Mtb is independent of CD36 and CERT1 gene expression (Fig. 2E and F).

### Myriocin reduces Mtb growth in macrophages involved in PPARγ pathway

**Fig 3:**
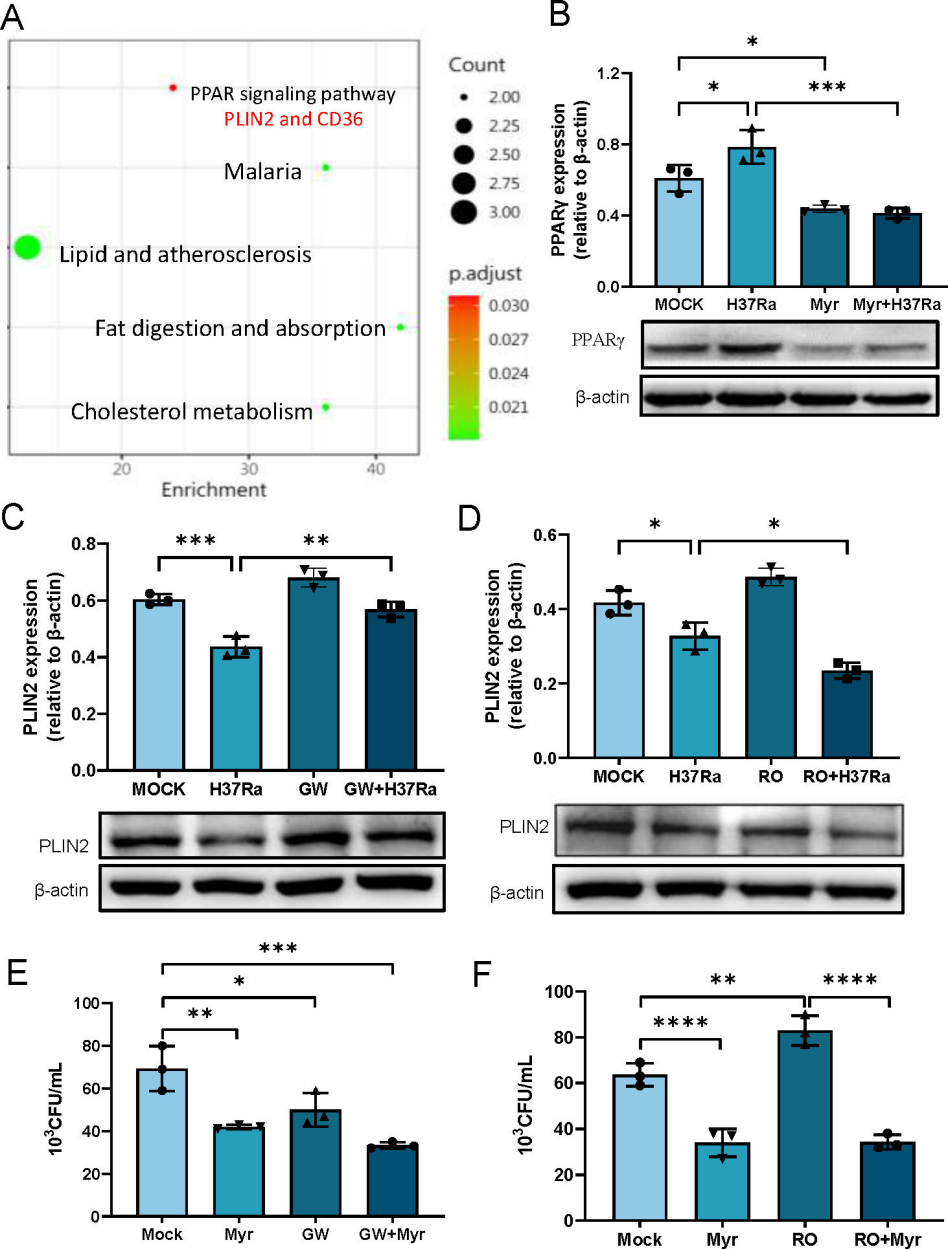
Myriocin reduces Mtb growth in macrophages involved in PPARγ pathway. (**A**) Dotplot of enriched terms, the plot shows enriched terms observed in myriocin treatment group during H37Ra infection. Size of the dot describes the number of DEGs belonging to the enriched terms, whereas the color represents the *P*-adjust value from the Over-Representation test. (**B**) PPARγ gene expression in mRNA and protein levels with or without myriocin treatment during H37Ra infection or not in PMA-differentiated THP-1 macrophages. Relative expression is normalized to β-actin, *n* = 3. (**C and D**) PLIN2 expression level was detected after treatment with GW9662 (**C**) or rosiglitazone (D) during H37Ra 24 h infection. (**E and F**) CFU assay was performed after treatment with GW9662 (**E**) or rosiglitazone (**F**) with or without myriocin during H37Ra 72 h infection, *n* = 3. Data are means ± S.E.M., *n* = 3. The differences among groups were compared using one-way ANOVA followed by Tukey’s multiple comparison test. *P < 0.05, **P < 0.01, ***P ≤ 0.001.

We further investigated the genes involved in lipid storage that may be regulated by myriocin during Mtb infection. We observed that PLIN2 and CD36 are involved in the PPARγ signaling pathway through gene set enrichment analysis (GSEA) (Fig. 3A). qRT-PCR and western blotting confirmed reduced expression of PPARγ at both mRNA and protein levels after myriocin treatment during Mtb infection (Fig. 3B). To investigate how PPARγ regulates PLIN2 gene expression, we pretreated cells with the PPARγ inhibitor GW9662 and the activator rosiglitazone. We detected PLIN2 expression at both mRNA and protein levels (Fig. 3C and D). PPARγ inhibition increased PLIN2 mRNA expression, while PPARγ activation decreased PLIN2 protein levels. Chromatin immunoprecipitation assays were conducted to assess the regulatory role of PPARγ on PLIN2. The results revealed that PPARγ can bind to the promoter region of PLIN2. Myriocin treatment increases the binding activity and consequently regulates gene expression (Fig. S5A and B). To assess the impact of the PPARγ signaling pathway on bacterial growth, we also performed a CFU assay after treatment with the PPARγ inhibitor GW9662 and the activator rosiglitazone (Fig. S6A and B). Inhibition of PPARγ significantly reduced Mtb growth in macrophages, while activation of PPARγ increased bacterial load (Fig. 3E and F).

### Myriocin increases the number of lipid droplets in macrophages infected with Mtb

**Fig 4:**
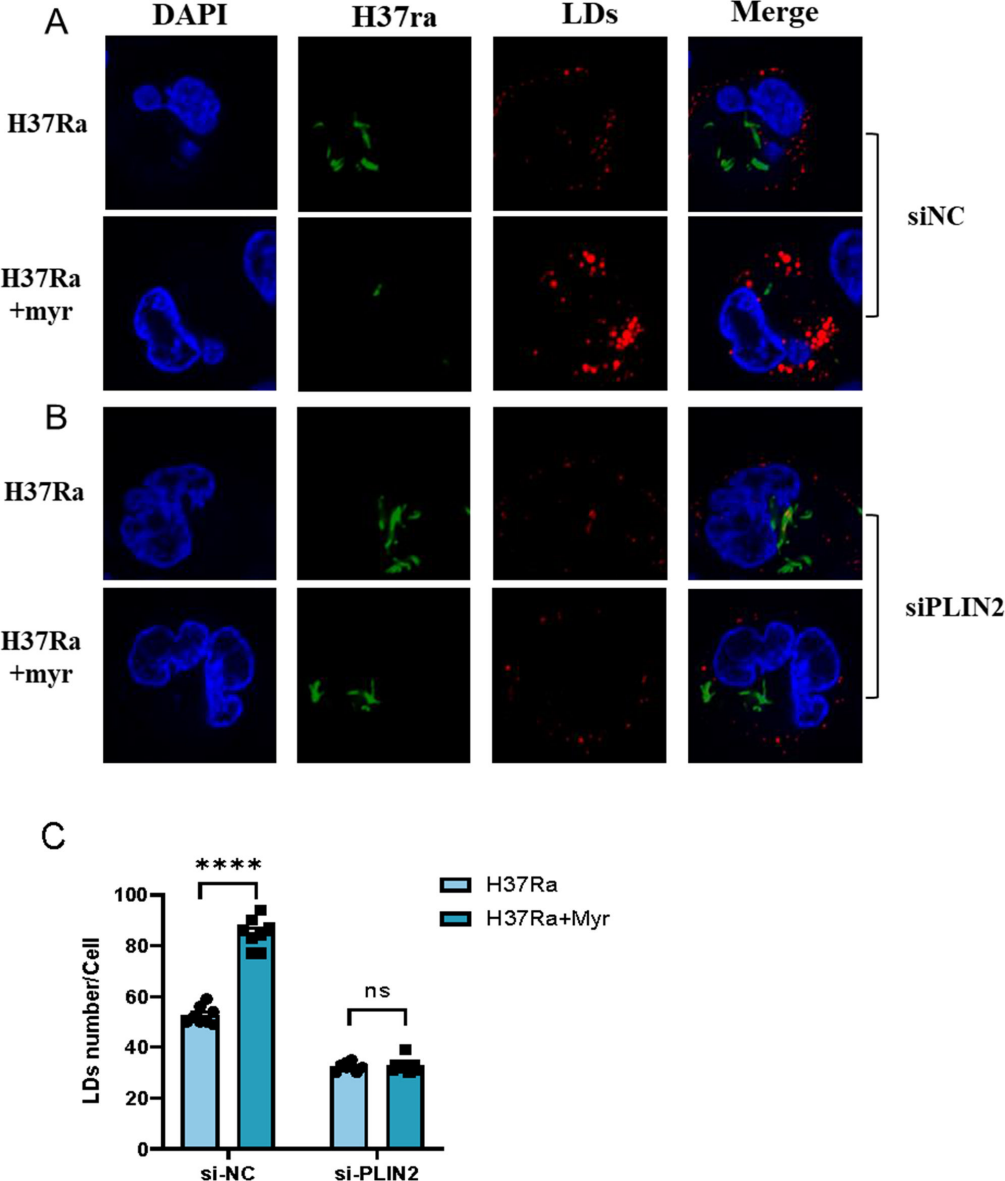
Myriocin increases the number of lipid droplets in macrophages during Mtb infection. PMA-differentiated THP-1 macrophages were transfected with control (A) or PLIN2 (B) siRNA for 48 h before infection with GFP-H37Ra (MOI  =  10:1) for another 6 h. Cells were treated with myriocin (5 µM) and incubated for an additional 24 h. Representative immunofluorescence confocal microscopy images (total 80–100 images) to illustrate the number of LDs (red) and Mtb (green) in macrophages; scale bars: 5 µm. Quantification of the co-localization using the merged area. Data are means ± S.E.M., *n* = 3. The differences among groups were compared using one-way ANOVA followed by Tukey’s multiple comparison test. *P < 0.05, **P < 0.01, ***P ≤ 0.001.

As PLIN2 gene expression was upregulated at 24 h post-myriocin treatment during Mtb infection (Fig. S7). To investigate the role of LDs in Mtb clearance by macrophages, we performed immunostaining and confocal microscopy experiments. We observed that macrophages treated with myriocin for 24 h exhibited a higher number and volume of LDs compared to those treated with DMSO (Fig. S8A). Silencing PLIN2 led to a significant decrease in the number and area of LDs (Fig. S8B). In line with our previous findings, myriocin treatment during H37Ra infection resulted in a reduction in Mtb load and an increase in the number of LDs in macrophages. However, when PLIN2 was silenced, this effect was reversed (Fig. 4A through C).

## DISCUSSION

Most individuals can suppress Mtb infection and prevent the development of active TB pathology by expressing a balanced metabolic immune response (2–4). The fate of macrophages plays a crucial role in the pathogenesis and host defense against Mtb (11). Macrophages employ various mechanisms to clear Mtb, including the generation of oxygen and nitrogen components, cytokines, acidification of phagosomes, and intracellular autophagy (4). However, Mtb has evolved strategies to survive within lipid-rich granulomas, which may be influenced by dysregulated host lipid production and storage induced by Mtb (7, 16). Recent studies have shed light on various roles of sphingomyelinases in manipulating mycobacterial infections. SCS has been found to promote the fusion of bacteria-containing phagosomes and lysosomes, while sphingomyelinase-derived ceramide induces cell death. Meanwhile, ceramide enhances the release of reactive oxygen species, which in turn suppresses autophagy in infected macrophages both *in vitro* and *in vivo* which allowing the pathogen to survive within macrophages. These findings highlight the significance of the SCS in the host’s defense against mycobacterial attacks. Drugs targeted on SCS might reverse the susceptibility to Mtb.

Our research found that myriocin treatment reduced both H37Rv and H37Ra bacterial load significantly. Myriocin is an effective inhibitor of SPT, which is crucial for *de novo* synthesis of sphingolipids and ceramides. we first hypothesize that the bacterial control by myriocin may involve reprogrammed ceramide metabolism. Ceramide is a central molecule involved in modulating membrane properties, plays a role in cellular processes such as apoptosis and inflammation. Inflammation is a host defense mechanism against Mtb stimuli (17, 18). Excessive pro-inflammatory responses during Mtb infection, regulated by lipid mediators (LMs) such as prostaglandins (PGs), leukotrienes (LTs), and cytokines, can lead to lung tissue necrosis, cavitation, and spread of Mtb (19). This enhanced inflammation causes lung tissue necrosis and cavitation, which contributes to the spread of Mtb (20, 21). This may be the other mechanism by which Mtb evades host killing. In line with other research, myriocin works by targeting the host immune response rather than directly attacking the bacteria. It has been found that myriocin modulates the host immune response, promoting an anti-inflammatory environment that can help control the excessive immune response often seen in TB. By doing so, myriocin may help prevent tissue damage and improve treatment outcomes. However, myriocin treatment only slightly reduces the upregulation of TNF but not expression of other inflammatory cytokines like IL-1B/IL-6/IL-10 (Fig. S9A through D and S10). The anti-inflammatory response regulated by myriocin may be mediated through lipid mediators. We will confirm this in our future research. We also conducted an experiment to investigate the regulatory effect of Myriocin on macrophage autophagy and apoptosis. The data revealed that pre-treatment with myriocin did not regulate autophagy and apoptosis in macrophages (Fig. S10 and S11).

To investigate gene regulation by myriocin during Mtb infection, we conducted RNA-seq analysis. The results showed a significant increase in PLIN2/CD36/CERT1 gene expression after myriocin treatment. Through GSEA, we found that PLIN2 and CD36 are involved in the PPARγ signaling pathway. PPARγ, as an important transcription factor, plays a crucial role in biological processes such as adipocyte differentiation, lipid metabolism, and inflammatory response. Through the regulation of gene expression, it is involved in the modulation of various physiological and pathological processes (22, 23). During Mtb infection, PPARγ knockdown or inhibition has been shown to significantly reduce Mtb growth in macrophages (15, 24). Interestingly, we also observed a decrease in the expression of PPARγ at both mRNA and protein levels following myriocin treatment during Mtb infection. Chromatin immunoprecipitation assay shows PPARγ could bind to the promoter region of PLIN2 (Fig. S7). Silencing PLIN2 and CD36 resulted in an increased bactericidal burden, whereas the bactericidal burden decreased after silencing the CERT1 gene, indicating their involvement in Mtb clearance. However, myriocin treatment did not affect bactericidal burden reduction solely by silencing PLIN2. All these findings indicate that myriocin-induced Mtb clearance in macrophages depends on the expression of LDs surface protein PLIN2. PLIN2 is a lipid droplet-associated protein that typically plays a role in regulating lipid metabolism and lipid droplet stability. In our research, myriocin upregulated PLIN2 expression leading to enhanced protein-mediated antimicrobial effects of lipid droplets, this may suggest a role for PLIN2 in immune responses. PLIN2 may mediate the transport of lipid droplets to mitochondria, leading to the reprogramming of lipid metabolism-immune crosstalk.

Host-derived lipids may support the life cycles of intracellular pathogens (25–27). LDs are the major lipid storage organelles that may provide the source of these lipids (28, 29). However, recent studies have shown that the creation of LDs in Mtb-infected macrophages inhibits bacterial uptake of host fatty acids while promoting the generation of protective lipid mediators derived from fatty acids (30, 31). During the course of *Mycobacterium* infection, our metabolomics data show that treatment with myriocin also alters the host’s lipid metabolism profile, leading to elevated levels of fatty acids, triglycerides, and other metabolic products. Among these, triglycerides are important components of lipid droplets, which may be a key factor contributing to the increase in lipid droplets. As a result, LDs might be the potential target to deliver effective host defenses against intracellular pathogens. PLIN2 is a protein associated with lipid droplets, plays a significant role in the formation, breakdown, and metabolism of lipid droplets. It is involved in the regulation of various physiological and pathological processes, including fat metabolism, obesity, and fatty liver, among others. Consistent with our findings, myriocin treatment inhibited PPARγ, upregulated PLIN2 expression, increased the number of LDs, and ultimately reduced bacterial load. However, a comprehensive understanding of PLIN2 remains an active area of research, and further studies are needed to uncover its intricate molecular mechanisms and biological functions.

Current TB treatment requires a long course of multiple antibiotics, which can lead to issues with patient adherence and the development of resistance. By targeting new mechanisms of drug resistance, novel treatments have the potential to be more effective in treating drug-resistant strains of TB, which are a significant challenge with current therapies. Our findings can contribute significantly to the development of novel treatments by providing insights into new drug targets. HDT based on myriocin may reduce off-target effects compared to broad-spectrum antibiotics used in current treatments, leading to fewer side effects for patients.

The main limitation of this article lies in the absence of a mouse model with an internal knockout of the PLIN2 gene. The mechanism by which PLIN2 regulates host metabolism and immunity still requires further research. while the current study provides a valuable theoretical basis for the therapeutic potential of myriocin in combination with other anti-TB drugs. By conducting more extensive clinical trials, mechanistic studies, and optimization of dosages, we can better understand and harness the potential synergistic effects of myriocin in combating tuberculosis. This research direction holds promise for developing more effective and efficient treatment strategies for TB in the future.

In conclusion, our findings reveal a novel mechanism by which myriocin enhances Mtb clearance in macrophages. By activating the surface protein PLIN2 of LDs, myriocin increases the number of LDs and their localization with Mtb. Myriocin may serve as a potential host-directed therapeutic strategy for TB.

## SUPPLEMENTAL MATERIAL

Supplemental material msphere.00257-24-s0001.pdf Fig. S1-S11.

ASM does not own the copyrights to Supplemental Material that may be linked to, or accessed through, an article. The authors have granted ASM a non-exclusive, world-wide license to publish the Supplemental Material files. Please contact the corresponding author directly for reuse.
