# Supplementary material for: Myriocin enhances the clearance of M. tuberculosis by macrophages through the activation of PLIN2
Source: mSphere. 2024 Jun 26;9(7):e00257-24. doi: 10.1128/msphere.00257-24 (PMC11288015; doi:10.1128/msphere.00257-24)
Supplement: Supplemental material — Fig. S1-S11. [file msphere.00257-24-s0001.pdf]

Supplementary Figure 1

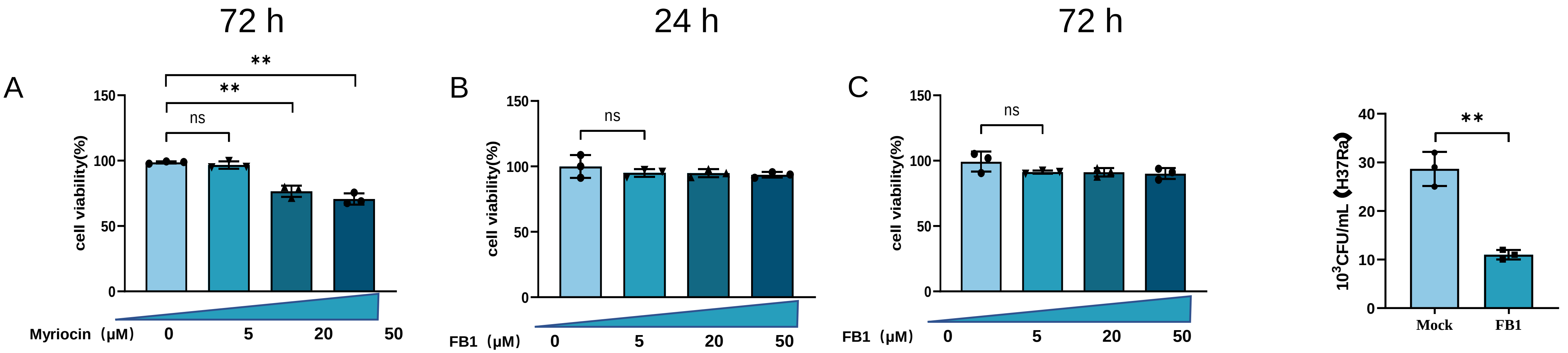

Supplementary Figure 1 (A)Effect of myriocin and FB1 on cell viability. PMA-differentiated THP-1 macrophages was treated with indicated concentration of FB1. After 24 h /72h incubation at 37 ℃, cell viability was assessed by CCK-8 assay. (B) PMA-differentiated THP-1 macrophages were infected with H37Ra (MOI of 10:1) for 6h. Then extracellular bacteria were removed, after 72h incubation with FB1, intracellular CFU was determined.

Supplementary Figure 2

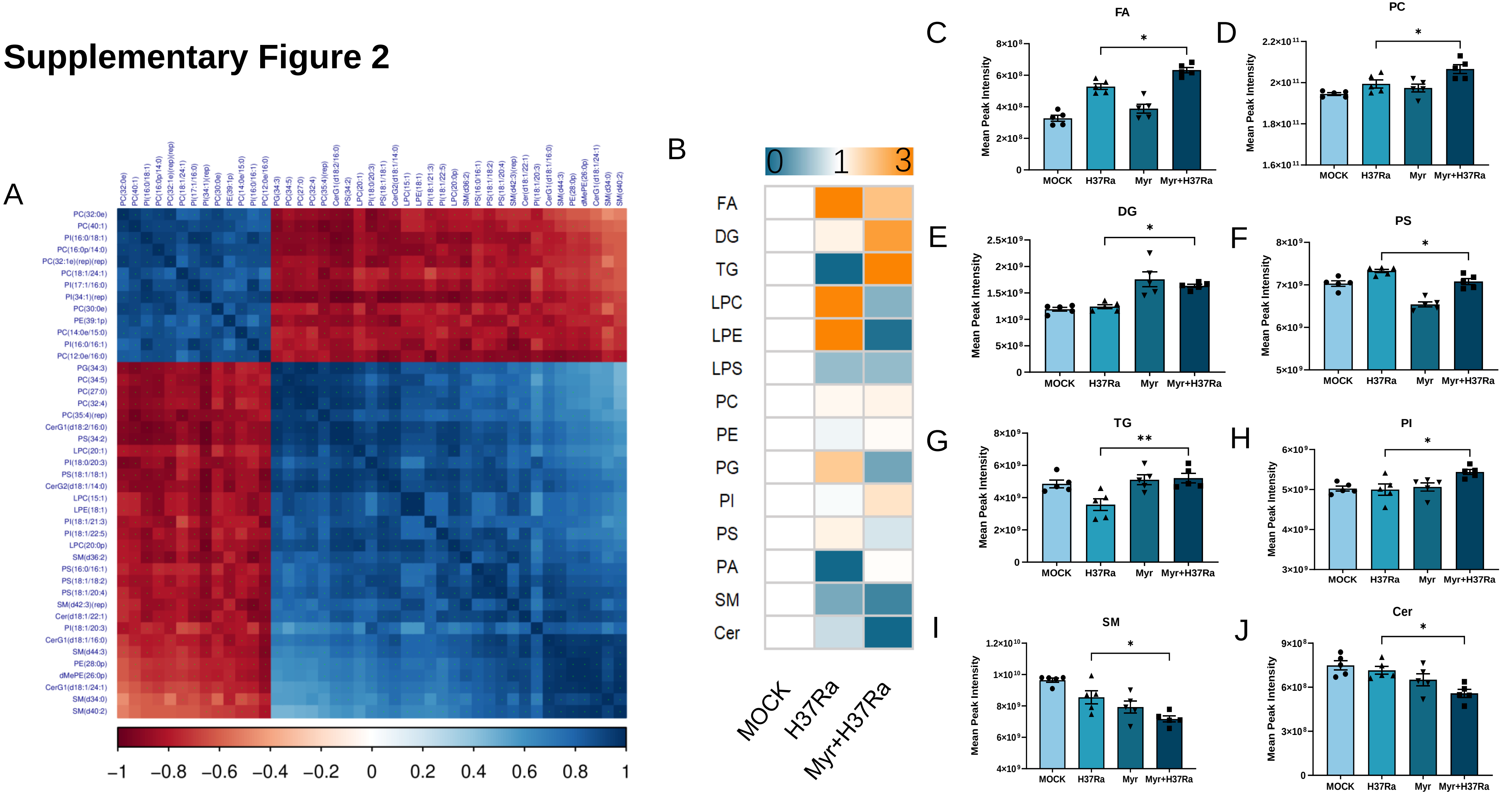

Supplementary Figure 2 PMA-differentiated THP-1 macrophages treated with or without myriocin during H37Ra infection.(A-B)Lipid metabolomics analysis in the supernatant was conducted. (C-H) In the process of Mtb infection, dysregulation of lipid metabolism products such as fatty acid(FA), Phosphatidylcholine(PC),Diacylglycerol(DG), Phosphatidylserine(PS), Triglycerides(TG), and phosphatidylinositol(PI) were detected. (I,J)Both Sphingomyelin (SM) and Ceramides (Cre) downregulated after myriocin treatment during Mtb infection.

Supplementary Figure 3

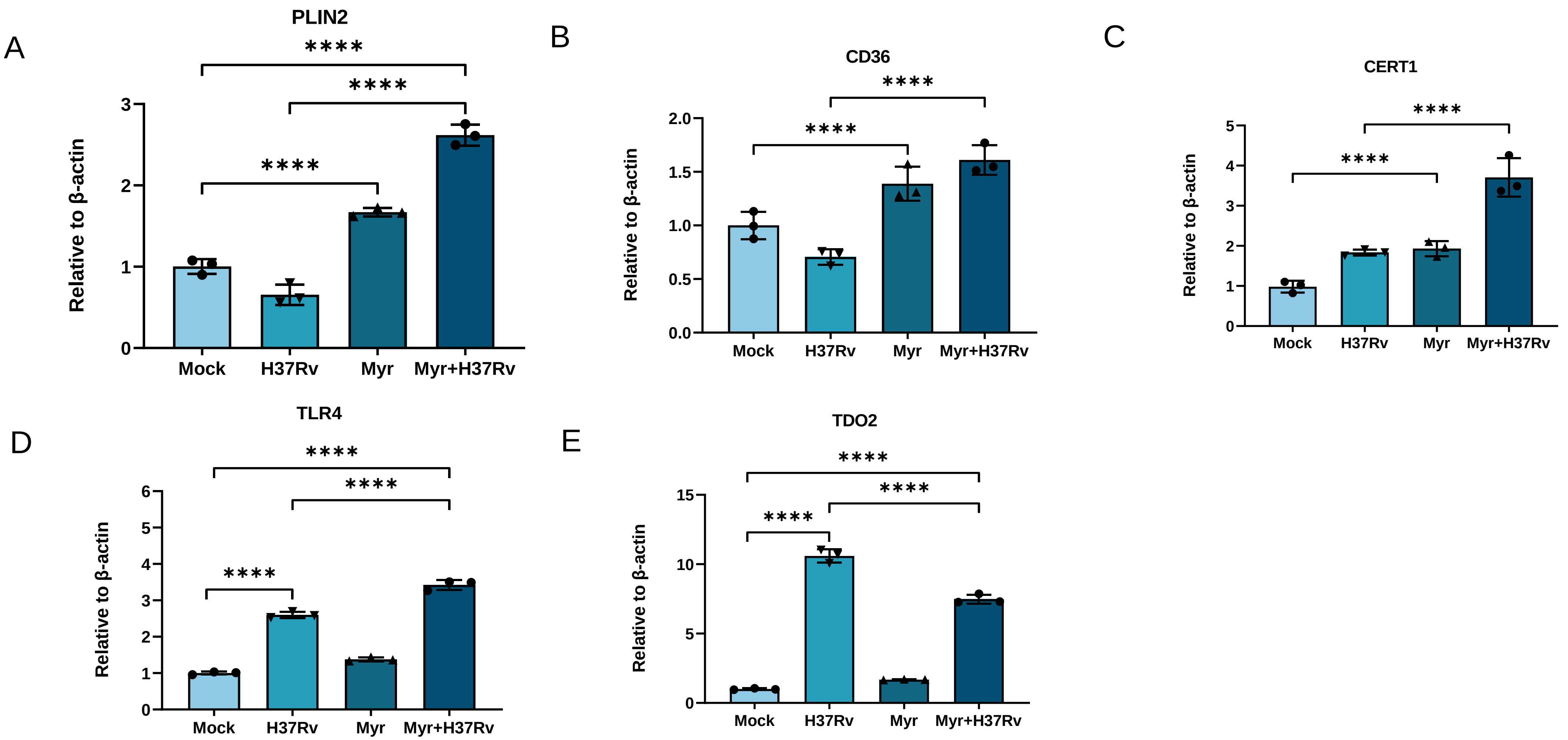

Supplementary Figure 3. Analysis of mRNA levels of PLIN2(A),CD36(B), CERT1(C), TLR4(D) TDO2(E) with or without myriocin treatment during H37Rv infection or not in PMA-differentiated THP-1 macrophages. Relative mRNA levels are normalized to β-actin, n=3.

Supplementary Figure 4

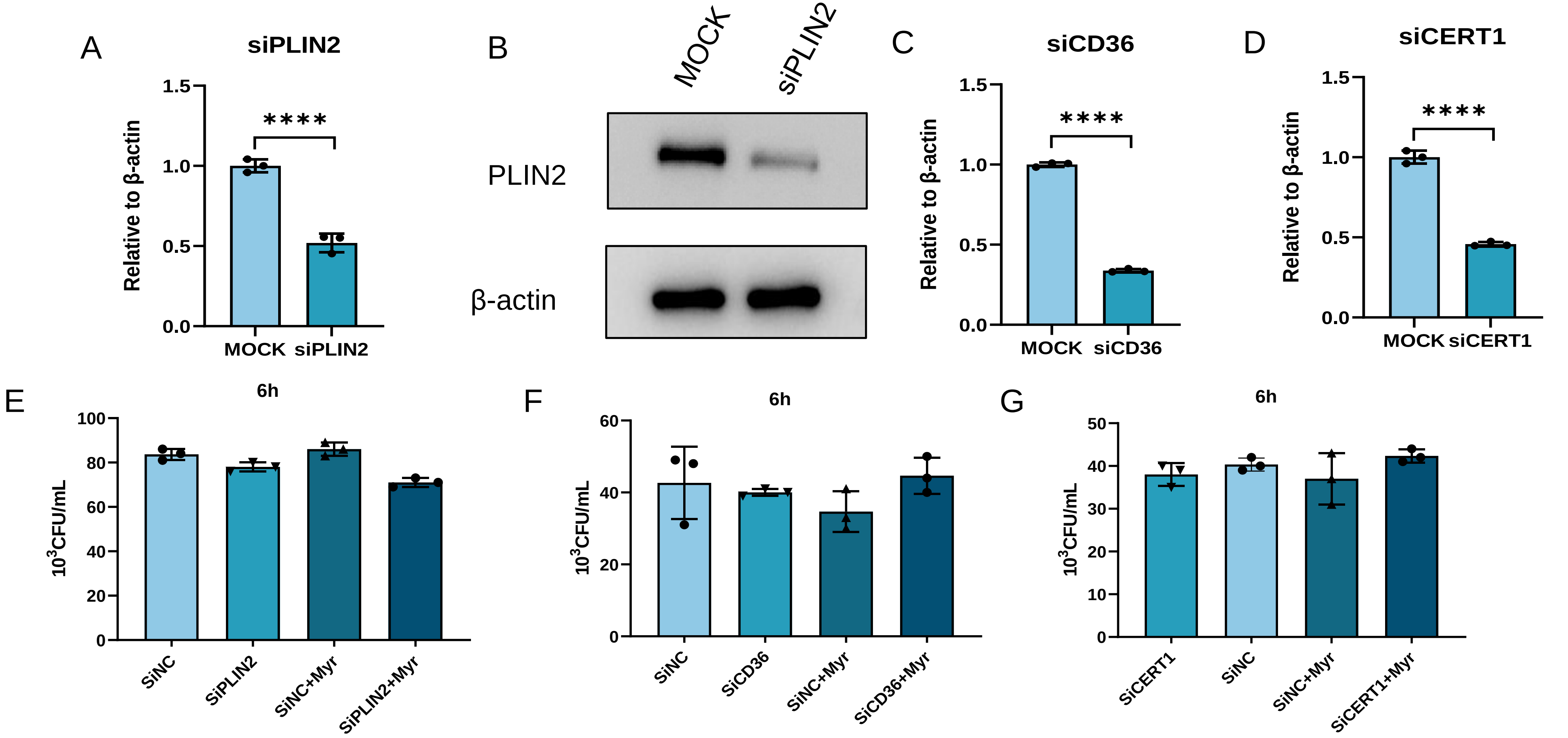

Supplementary Figure 4 (A-D) THP-1 were transfected with siPLIN2, siCD36 and siCERT1 or scrambled control siRNA (MOCK), representative RT-PCR showing knockdown efficiency (n=3). (D-G) CFU assay was performed after silenced PLIN2 (E), CD36 (F) and CERT1(G) with or without myriocin treatment during H37Ra 6h infection, n=3.

Supplementary Figure 5

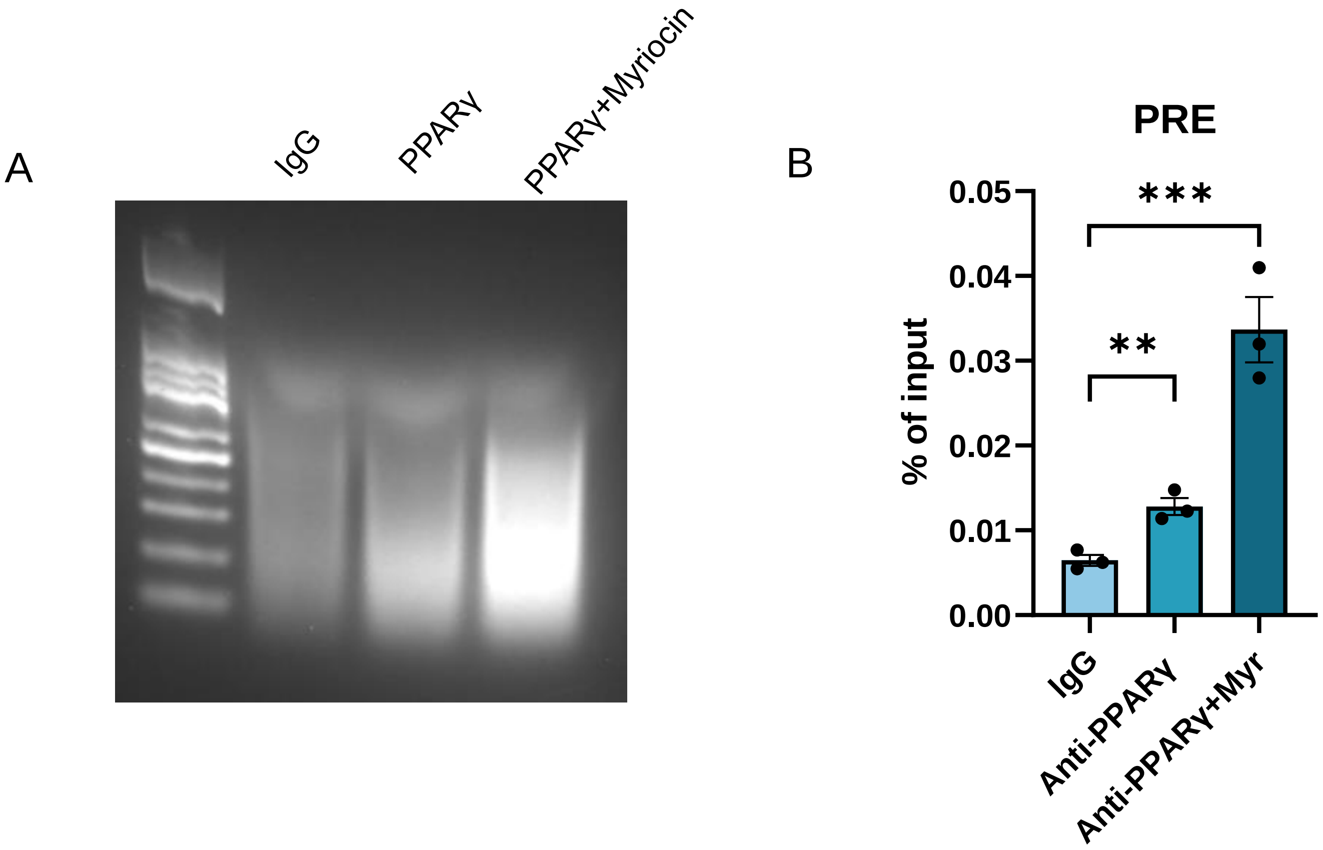

Supplementary Figure 5 (A,B)PPARγ can bind to the response element of the PLIN2 gene. Myriocin treatment increase the binding activity.

Supplementary Figure 6

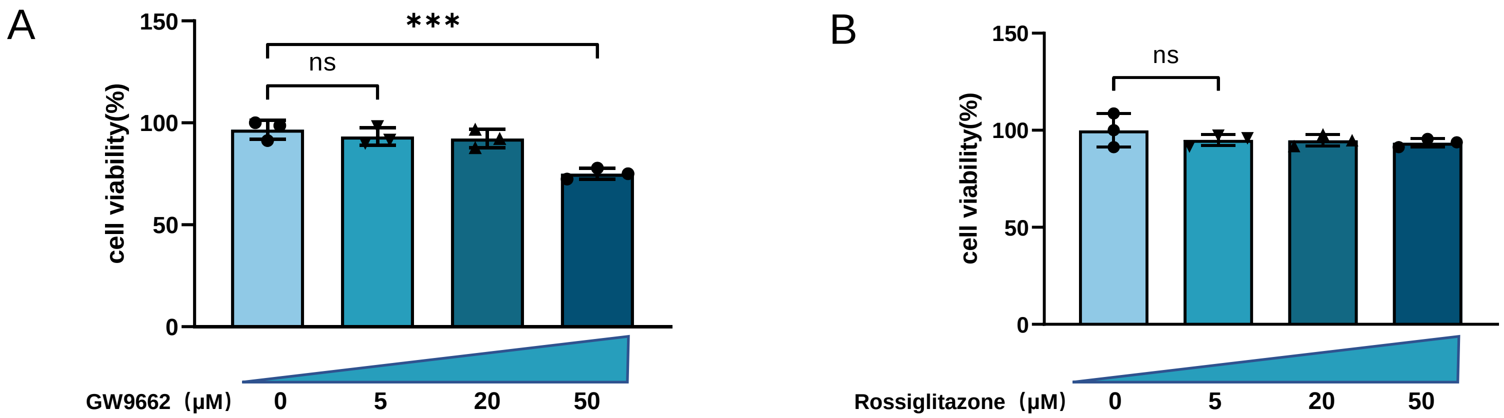

Supplementary Figure 6 Effect of GW9662 or rossiglitazone on cell viability. PMA-differentiated THP-1 macrophages was treated with indicated concentration of GW9662 or rossiglitazone. After 24 h incubation at 37 ℃, cell viability was assessed by CCK-8 assay.

Supplementary Figure 7

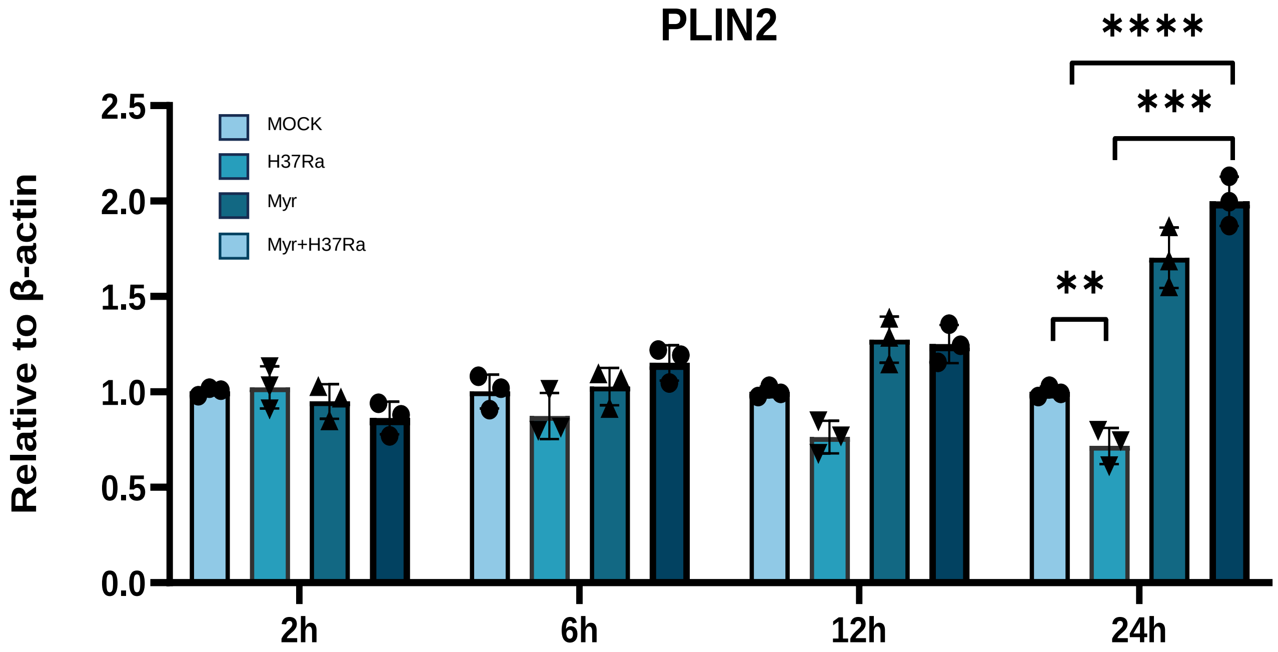

Supplementary Figure 7 MDMs were infected with H37Ra and treated with myriocin for 2h,6h,12h and 24h. PLIN2 gene expression was detected in different time point. Relative mRNA levels are normalized to β-actin, n=3.

Supplementary Figure 8

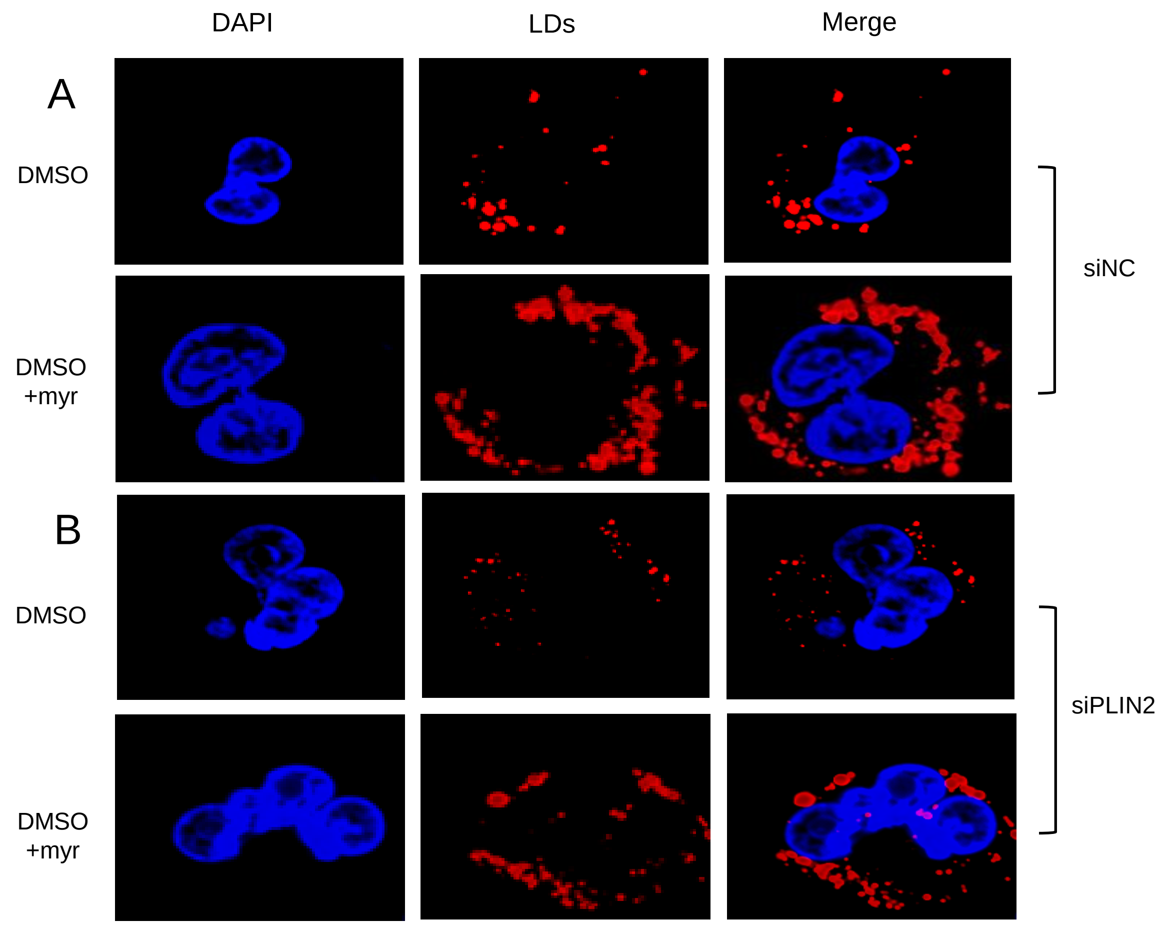

Supplementary Figure 8 Representative immunofluorescence confocal microscopy images (total 80 to 100 images) to illustrate the staining of LDs with myriocin treatment in siNC or siPLIN2 macrophages; scale bars: 5 μm.

Supplementary Figure 9

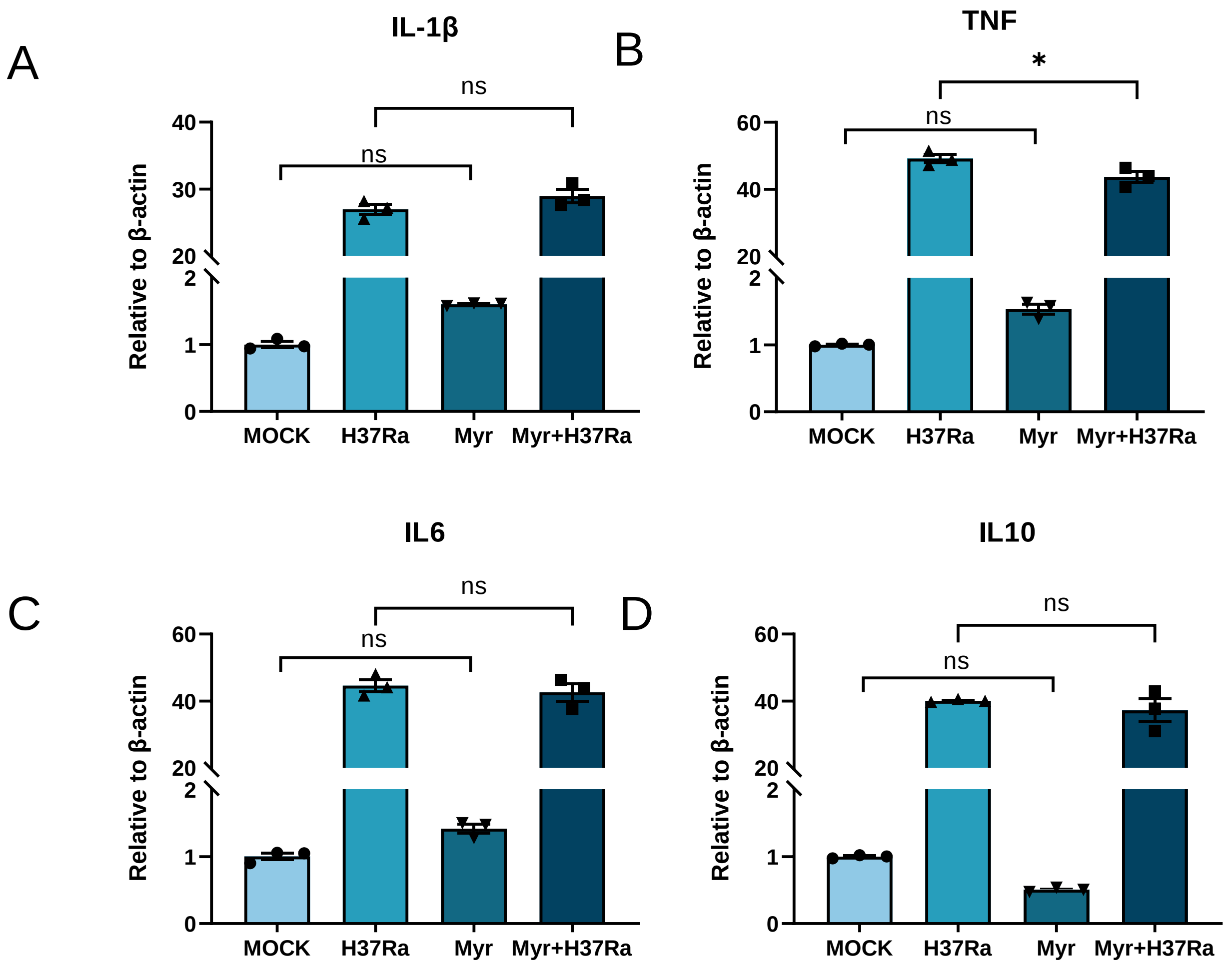

Supplementary Figure 9 Myriocin treatment reduce TNF expression induced by Mtb infection. Analysis of mRNA levels of IL1 (A),TNF(B), IL6(C), IL10(D) with or without myriocin treatment during H37Ra infection or not in PMA-differentiated THP-1 macrophages. Relative mRNA levels are normalized to β-actin, n=3.

Supplementary Figure 10

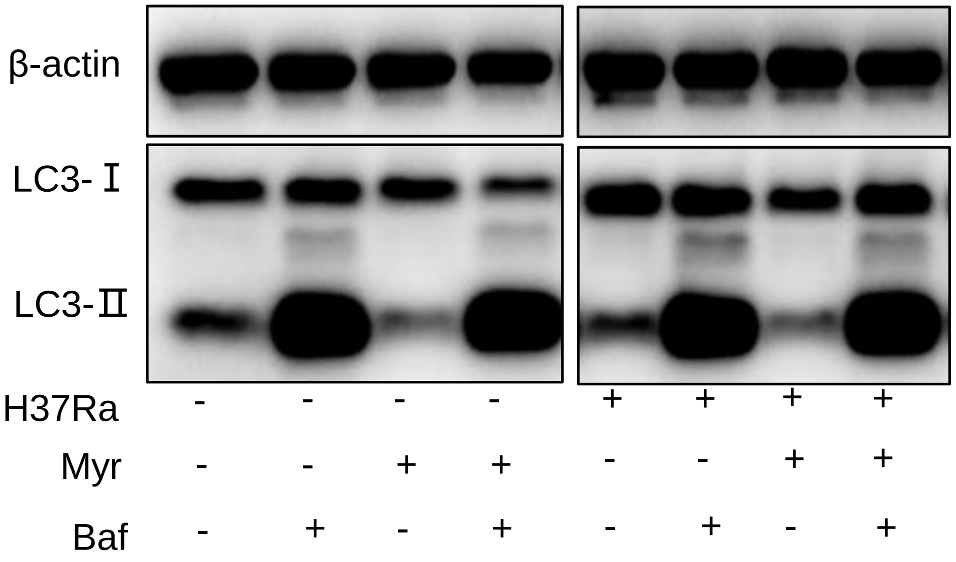

Supplementary Figure 11 Myriocin treatment did not influent autophage induced by Mtb infection.

Supplementary Figure 11

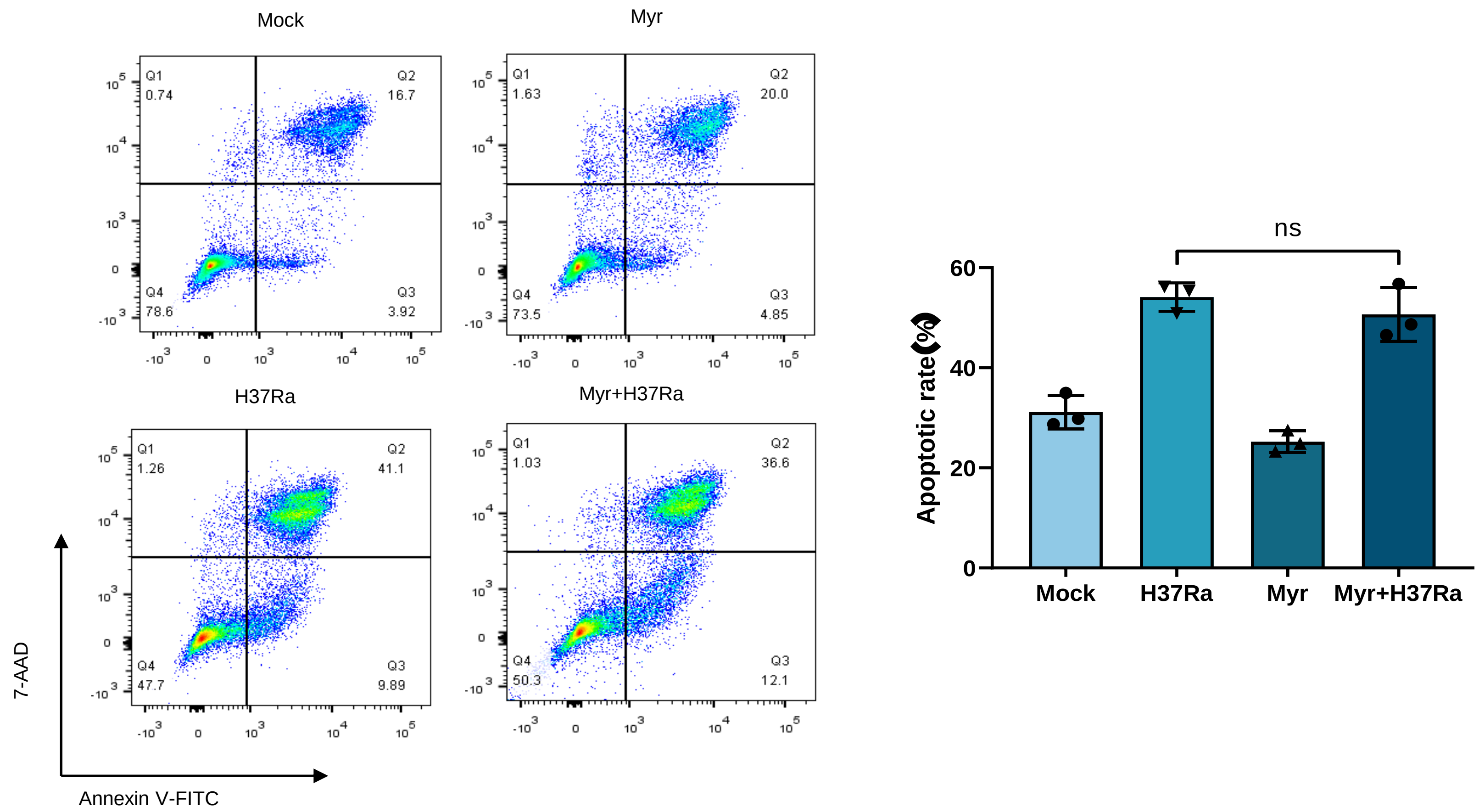

Supplementary Figure 11 Myriocin treatment did not reduce the apoptosis rate induced by Mtb infection.
